# Supplementary material for: Hybrid Nanomaterial of Graphene Oxide Quantum Dots with Multi-Walled Carbon Nanotubes for Simultaneous Voltammetric Determination of Four DNA Bases
Source: Nanomaterials (Basel). 2023 Apr 29;13(9):1509. doi: 10.3390/nano13091509 (PMC10180489; doi:10.3390/nano13091509)
Supplement: Supplementary file 1 [file nanomaterials-13-01509-s001.zip › nanomaterials-2341560-supplementary.pdf]

nanomaterials

Supplementary Information

Hybrid Nanomaterial of Graphene Oxide Quantum Dots with Multi-Walled Carbon Nanotubes for Simultaneous Voltammetric Determination of Four DNA Bases

Qusai Hassan [1,2,†], Chevon Riley [1,†], Meissam Noroozifar [1,*] and Kagan Kerman [1,2,*]

1 Department of Physical and Environmental Sciences, University of Toronto Scarborough, 1265 Military Trail, Toronto, ON M1C 1A4, Canada; qusai.hassan@mail.utoronto.ca (Q.H.); chevon.riley@mail.utoronto.ca (C.R.)
2 Department of Chemistry, University of Toronto, 80 St. George Street, Toronto, ON M5S 3H6, Canada
* Correspondence: m.noroozifar@utoronto.ca (M.N.), kagan.kerman@utoronto.ca (K.K.)
† These authors contributed equally to the work.

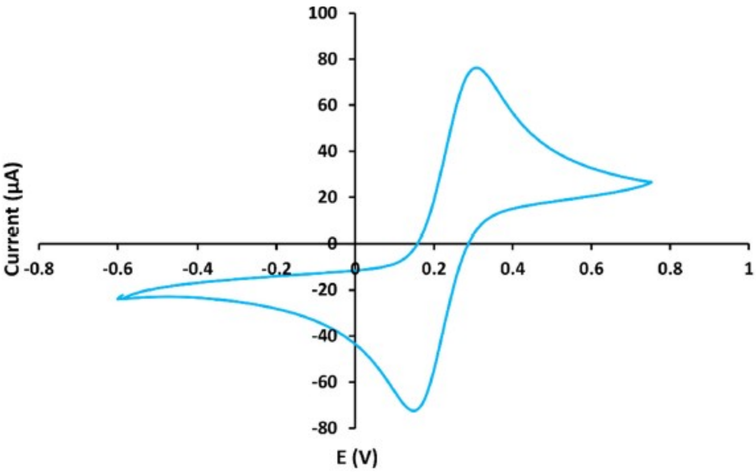

Figure S1. Cyclic voltammogram of a bare GCE in a solution of 5 mM of both $K_3[Fe(CN)_6]$ and $K_4[Fe(CN)_6]$ with 100 mM of KCl in 0.2 M PBS (pH 7.0). The formal potential was estimated as 0.23 V *vs.* Ag/AgCl (using $E_f = (E_{anodic} + E_{cathodic})/2$), where E_{anodic} = 0.30 V and $E_{cathodic}$ = 0.15 V.

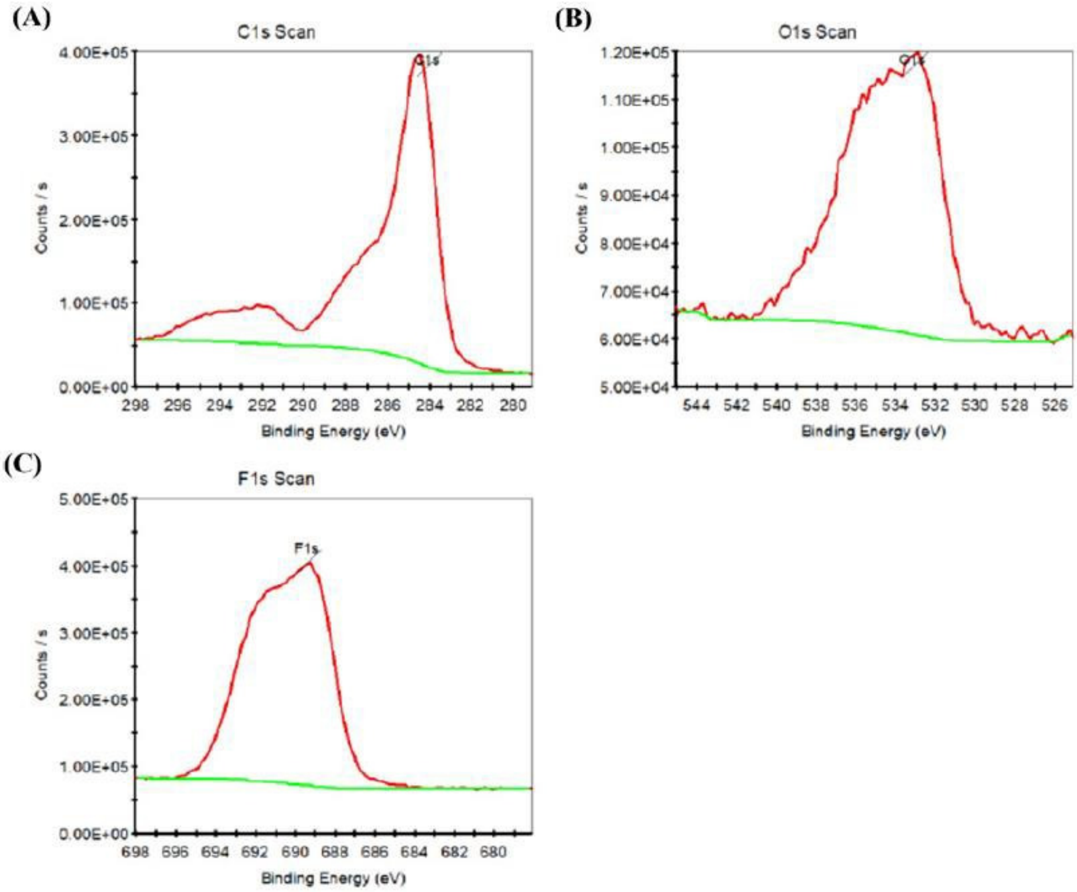

Figure S2. XPS spectra of **(A)** carbon, **(B)** oxygen, and **(C)** fluorine. The red spectra refer to the experimental acquisition while the green spectra refer to the background acquisition.

(A)

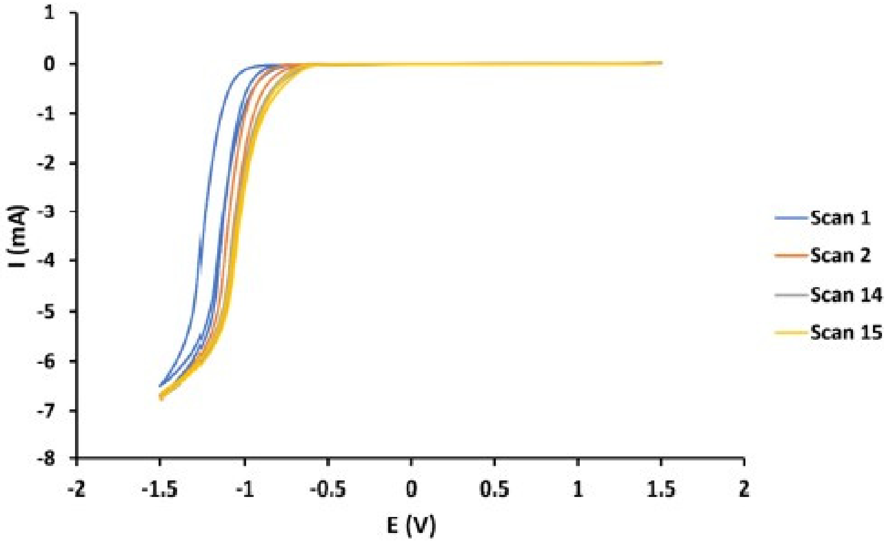

(B)

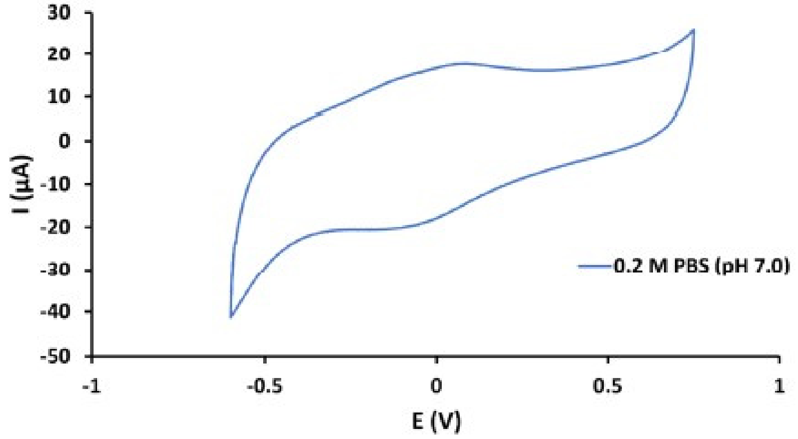

Figure S3. (A) Cyclic voltammograms showing the cleaning of the bare GCE in 1 M H_2SO_4 at a scan rate of 100 mV/s. The first two and last two scans are shown. **(B)** Cyclic voltammogram of a bare GCE in 0.2 M PBS (pH 7.0) at a scan rate of 1 V/s. The electrode capacity values calculated for the oxidation and reduction peak were 1.63×10^{-7} C and 2.33×10^{-6} C, respectively.

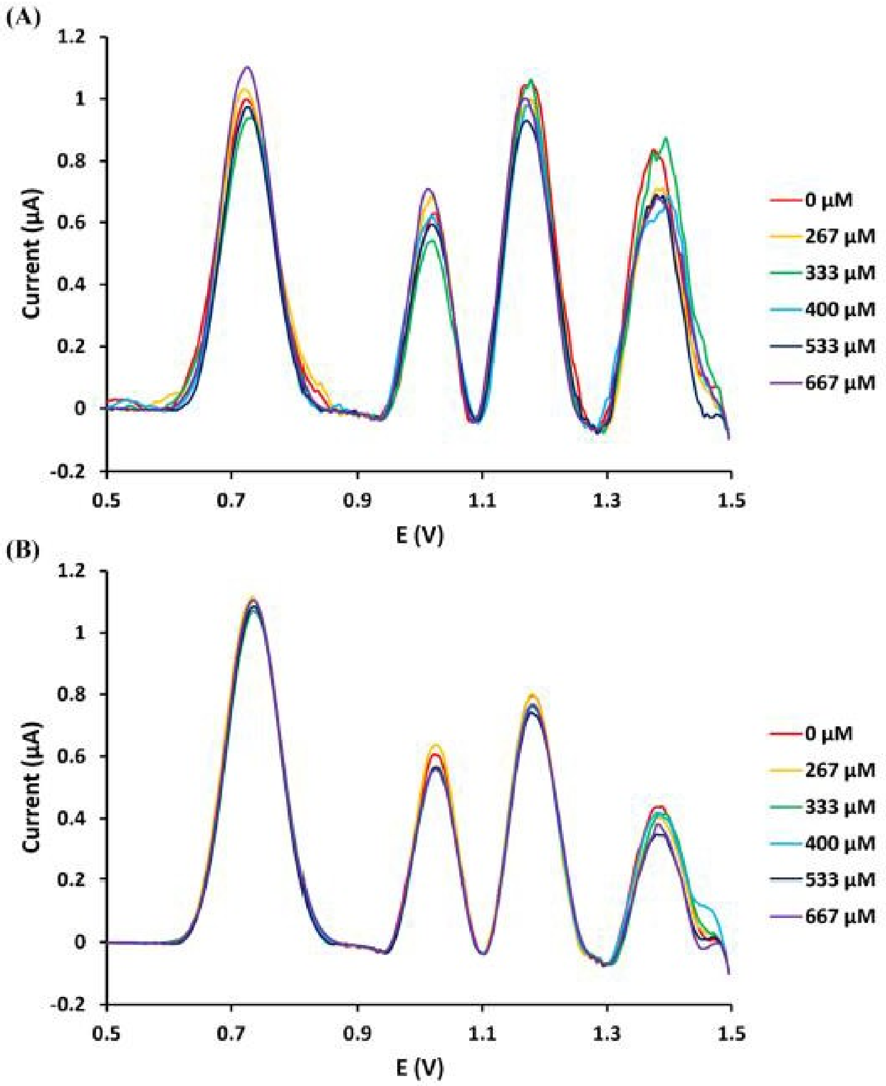

Figure S4. Interference study of G (40 μM), A (40 μM), T (400 μM), and C (400 μM) while varying the concentrations of **(A)** ascorbic acid and **(B)** glucose from 267 to 667 μM at GOQD-MWCNT/GCE in 0.2 M PBS (pH 7.0).
